# Relatedness of type IV pilin PilA amongst geographically diverse *Moraxella bovoculi* isolated from cattle with infectious bovine keratoconjunctivitis

**DOI:** 10.1099/jmm.0.001293

**Published:** 2021-01-06

**Authors:** John A. Angelos, Kristin A. Clothier, Regina L. Agulto, Boguslav Mandzyuk, Morten Tryland

**Affiliations:** ^1^​ Department of Medicine & Epidemiology, School of Veterinary Medicine, University of California, Davis, CA 95616, USA; ^2^​ Department of Pathology, Microbiology & Immunology, School of Veterinary Medicine, University of California, Davis, CA 95616, USA; ^3^​ Department of Arctic and Marine Biology, UiT The Arctic University of Norway, Framstredet 39, N-9037 Tromsø, Norway

**Keywords:** infectious bovine keratoconjunctivitis, *Moraxella bovis*, *Moraxella bovoculi*, pilin, PilA, pinkeye

## Abstract

**Introduction:**

*
Moraxella bovoculi* is frequently isolated from the eyes of cattle with infectious bovine keratoconjunctivitis (IBK; pinkeye). As with *M. bovis,* which has been causally linked to IBK, *M. bovoculi* expresses an RTX (repeats in the structural toxin) cytotoxin that is related to *M. bovis* cytotoxin. Pilin, another pathogenic factor in *M. bovis *, is required for corneal attachment. Seven antigenically distinct pilin serogroups have been described in *M. bovis *.

**Hypothesis/Gap Statement:**

Multiple different serogroups exist amongst type IV pilin encoded by *M. bovis *, however, it is not known whether *M. bovoculi* exhibits a similar degree of diversity in type IV pilin that it encodes.

**Aim:**

This study was done to characterize a structural pilin (PilA) encoded by *M. bovoculi* isolated from cases of IBK to determine if diversity exists amongst PilA sequences.

**Methodology:**

Ninety-four isolates of *M. bovoculi* collected between 2002 and 2017 from 23 counties throughout California and from five counties in four other Western states were evaluated.

**Results:**

DNA sequencing and determination of deduced amino acid sequences revealed ten (designated groups A through J) unique PilA sequences that were ~96.1–99.3 % identical. Pilin groups A and C matched previously reported putative PilA sequences from *M. bovoculi* isolated from IBK-affected cattle in the USA (Virginia, Nebraska, and Kansas) and Asia (Kazakhstan). The ten pilin sequences identified were only ~74–76 % identical to deduced amino acid sequences of putative pilin proteins identified from the previously reported whole-genome sequences of *M. bovoculi* derived from deep nasopharyngeal swabs of IBK-asymptomatic cattle.

**Conclusions:**

Compared to the diversity reported between structural pilin proteins amongst different serogroups of *M. bovis *, *M. bovoculi* PilA from geographically diverse isolates derived from IBK-affected cattle are more conserved.

## Data Summary

Accession numbers for all supporting sequence data are provided in Table 1.

**Table 1.** Summary of GenBank Accession numbers for PilA and ISR sequences in 94 isolates of *M. bovoculi* isolated from cattle with infectious bovine keratoconjunctivitis

GenBank PilA Accession No.	GenBank ISR Accession No.	Collected by*	Collection year	PilA group	Isolate	County locations in the USA
MT333648	MT353781	JAA	2002	A	8342	Yuba County, CA
MT333649	MT353782	JAA	2002	A	6170	Yuba County, CA
MT333650	MT353783	JAA	2002	A	4794	Yuba County, CA
MT333651	MT353784	JAA	2002	A	4787	Yuba County, CA
MT333652	MT353785	JAA	2002	A	4786	Yuba County, CA
MT333653	MT353786	JAA	2002	F	4785	Yuba County, CA
MT333654	MT353787	JAA	2002	A	4773	Yuba County, CA
MT333655	MT353788	JAA	2002	A	2473	Yuba County, CA
MT333656	MT353789	JAA	2002	B	2470–1	Yuba County, CA
MT333657	MT353790	JAA	2002	A	2467	Yuba County, CA
MT333658	MT353791	JAA	2002	I	380	Yuba County, CA
MT333659	MT353792	JAA	2002	A	376	Yuba County, CA
MT333660	MT353793	JAA	2002	A	371	Yuba County, CA
MT333661	MT353794	JAA	2002	A	317	Yuba County, CA
MT333662	MT353795	JAA	2002	A	237	Yuba County, CA
MT333663	MT353796	JAA	2006	B	153R	Yuba County, CA
MT333664	MT353797	JAA	2006	B	151RB	Yuba County, CA
MT333665	MT353798	JAA	2008	B	130LB	Yuba County, CA
MT333666	MT353799	JAA	2008	B	112R	Yuba County, CA
MT333667	MT353800	JAA	2006	A	111L	Yuba County, CA
MT333668	MT353801	JAA	2007	B	108RB	Yuba County, CA
MT333669	MT353802	JAA	2007	A	108LB	Yuba County, CA
MT333670	MT353803	JAA	2008	C	105L	Yuba County, CA
MT333671	MT353804	JAA	2006	C	78LB	Yuba County, CA
MT333672	MT353805	JAA	2007	B	67LB	Yuba County, CA
MT333673	MT353806	KAC	2017	E	60	Kings County, CA
MT333674	MT353807	JAA	2007	A	59RB	Yuba County, CA
MT333675	MT353808	JAA	2007	B	59LB	Yuba County, CA
MT333676	MT353809	KAC	2017	A	59	Franklin County, ID
MT333677	MT353810	KAC	2016	H	58	Tulare County, CA
MT333678	MT353811	KAC	2016	D	57	Kern County, CA
MT333679	MT353812	KAC	2016	A	56	Tulare County, CA
MT333680	MT353813	KAC	2017	A	55	Kings County, CA
MT333681	MT353814	KAC	2017	C	54	Kern County, CA
MT333682	MT353815	KAC	2017	A	53	Tulare County, CA
MT333683	MT353816	KAC	2013	C	51	Yolo County, CA
MT333684	MT353817	KAC	2017	B	50	Jerome County, ID
MT333685	MT353818	KAC	2017	B	49	Stanislaus County, CA
MT333686	MT353819	KAC	2017	A	48	Placer County, CA
MT333687	MT353820	KAC	2017	A	47	Humboldt County, CA
MT333688	MT353821	JAA	2007	C	46L	Yuba County, CA
MT333689	MT353822	KAC	2011	A	46	Merced County, CA
MT333690	MT353823	KAC	2011	A	45	Stanislaus County, CA
MT333691	MT353824	KAC	2011	A	44	Modoc County, CA
MT333692	MT353825	KAC	2011	A	43	Siskiyou County, CA
MT333693	MT353826	KAC	2011	E	42	Sonoma County, CA
MT333694	MT353827	JAA	2008	A	41LB	Yuba County, CA
MT333695	MT353828	KAC	2011	B	41	San Joaquin County, CA
MT333696	MT353829	KAC	2012	A	40	Merced County, CA
MT333697	MT353830	KAC	2011	A	39	Yuba County, CA
MT333698	MT353831	KAC	2013	B	38	Marin County, CA
MT333699	MT353832	KAC	2014	D	37	Lassen County, CA
MT333700	MT353833	KAC	2014	A	36	Humboldt County, CA
MT333701	MT353834	KAC	2012	C	35	Sonoma County, CA
MT333702	MT353835	KAC	2012	B	34	Trinity County, CA
MT333703	MT353836	JAA	2006	B	33RB	Yuba County, CA
MT333704	MT353837	KAC	2013	C	33	Yuba County, CA
MT333705	MT353838	KAC	2012	C	32	Modoc County, CA
MT333706	MT353839	JAA	2008	A	31L	Yuba County, CA
MT333707	MT353840	KAC	2013	A	31	Yavapai County, AZ
MT333708	MT353841	JAA	2006	B	30LB	Yuba County, CA
MT333709	MT353842	KAC	2013	A	30	El Dorado County, CA
MT333710	MT353843	JAA	2007	B	29RB	Yuba County, CA
MT333711	MT353844	KAC	2013	A	29	Merced County, CA
MT333712	MT353845	KAC	2012	A	28	Chaves County, NM
MT333713	MT353846	KAC	2012	J	27	Merced County, CA
MT333714	MT353847	JAA	2007	B	26RB	Yuba County, CA
MT333715	MT353848	KAC	2013	A	26	Mendocino County, CA
MT333716	MT353849	KAC	2012	C	25	Marin County, CA
MT333717	MT353850	KAC	2013	A	24	Marin County, CA
MT333718	MT353851	KAC	2017	A	23	Modoc County, CA
MT333719	MT353852	KAC	2014	A	22	San Bernardino County, CA
MT333720	MT353853	KAC	2017	A	21	Kern County, CA
MT333721	MT353854	KAC	2015	A	20	Fresno County, CA
MT333722	MT353855	KAC	2017	A	19	Yolo County, CA
MT333723	MT353856	JAA	2006	B	18LB	Yuba County, CA
MT333724	MT353857	KAC	2015	A	18	Plumas County, CA
MT333725	MT353858	KAC	2015	C	17	Calaveras County, CA
MT333726	MT353859	KAC	2015	G	16	Yolo County, CA
MT333727	MT353860	KAC	2015	C	15	Sonoma County, CA
MT333728	MT353861	KAC	2016	D	14	Merced County, CA
MT333729	MT353862	KAC	2016	D	13	Merced County, CA
MT333730	MT353863	KAC	2015	A	12	Humboldt County, CA
MT333731	MT353864	KAC	2017	D	11	Merced County, CA
MT333732	MT353865	KAC	2008	C	10	Merced County, CA
MT333733	MT353866	KAC	2008	A	9	Sonoma County, CA
MT333734	MT353867	KAC	2008	A	8	Merced County, CA
MT333735	MT353868	KAC	2009	B	7	Sacramento County, CA
MT333736	MT353869	KAC	2009	C	6	Sonoma County, CA
MT333737	MT353870	KAC	2009	A	5	Merced County, CA
MT333738	MT353871	KAC	2009	A	4	Merced County, CA
MT333739	MT353872	KAC	2010	C	3	Stanislaus County, CA
MT333740	MT353873	KAC	2009	A	2	Merced County, CA
MT333741	MT353874	KAC	2010	A	1	Washoe County, NV

*JAA: John A. Angelos; KAC: Kristin A. Clothier

**Impact Statement**

Pilin (PilA) from *M. bovoculi* is conserved amongst geographically diverse isolates derived from cattle with IBK and displays considerably less variability amongst isolates compared to *M. bovis* pilins from different *M. bovis* serogroups. The significance of *M. bovoculi* pilin as it relates to the pathogenesis of IBK is presently unknown.

## Introduction

Infectious bovine keratoconjunctivitis (IBK; pinkeye) is the most common eye disease of cattle and is characterized by the presence of corneal ulceration, corneal oedema, conjunctivitis, and eye pain. First reported in 2007 [1], *M. bovoculi* is now more frequently isolated from eyes of cattle affected with IBK compared to *M. bovis* [2, 3]. While Koch’s postulates were previously established for *M. bovis* and IBK [4], a direct link between the type strain of *M. bovoculi* (no. 237) and corneal ulceration in a scarification model of infection in dairy calves could not be established [5]. Two distinct genotypes have been characterized in *M. bovoculi *; genotype 1 is associated with IBK-affected cattle while genotype 2 is associated with IBK-asymptomatic cattle [6, 7]. Recent studies have also identified different matrix-assisted laser desorption/ionization time-of-flight mass spectrometry (MALDI-TOF MS) profiles between the two genotypes [8].

From reports of isolations of *M. bovoculi* in different parts of the world, it is known that this organism has widespread geographic distribution amongst cattle as well as other ruminant [9, 10] and non-ruminant [11] species. One specific pathogenic factor that may be important in the capacity for *M. bovoculi* to contribute to the pathogenesis of IBK is an RTX (repeats in the structural toxin) toxin [12] that has been shown to be similar to the *M. bovis* RTX toxin (cytotoxin) [13]. The role of this RTX toxin in the pathogenesis of *M. bovoculi* is not known and some genotype 1 strains have been identified that do not possess RTX toxin genes [6, 7].

In addition to cytotoxin, the pathogenesis of *M. bovis* involves expression of pili that allow it to adhere to corneal epithelial cells [14–16]. For *M. bovis* it is thought that the presence of multiple *M. bovis* pilus serogroups [17] coupled with pilin gene inversions [18] increases antigenic variability and accounts for antigenic switching that may allow *M. bovis* to evade a host’s immune response [19].

The purpose of this study was to characterize PilA from geographically diverse Western USA isolates of *M. bovoculi* from IBK-affected cattle. We also sought to compare deduced pilin amino acid sequences from these Western USA isolates with pilin-related sequences from isolates of *M. bovoculi* whose full-genome sequences were previously submitted to GenBank, pilin-related sequences from *M. ovis,* and previously defined pilins from characterized serogroups of *M. bovis *.

## Methods

### Bacterial isolate source and identification

A total of 94 isolates of *M. bovoculi* from cases of IBK in cattle from 28 counties were used for this study (see Table 1). Bacterial isolates were cultured from ocular swabs from eyes of cattle with IBK that had been collected by one of the authors (JAA), or submitted to the California Animal Health and Food Safety Laboratory (CAHFS), Davis, CA and provided by one of the authors (KAC). Isolates (*n*=35) collected by JAA were from beef calves at the University of California Sierra Foothills Research and Extension Center, Brown’s Valley, Yuba County, CA (SFREC) during 2002 (*n*=5), 2006 (*n*=7), 2007 (*n*=8) and 2008 (*n*=5), or from IBK-affected dairy calves at a commercial dairy in Yuba County, CA during 2002 (*n*=10). Isolates provided by KAC (*n*=59) originated from cattle in 23 California counties (*n*=54), 2 Idaho counties (*n*=2), Arizona (*n*=1), Nevada (*n*=1), and New Mexico (*n*=1) during 2008–2017. Amongst the SFREC isolates collected during 2008, two isolates originated from each of two steers that had developed a corneal ulcer associated with IBK in a left eye, recovered and then developed an ulcer associated with IBK in the right eye 4–10 weeks later. One isolate included in this study from JAA (* M. bovoculi* 237) is the type strain for the species [1].

Isolates were confirmed as *M. bovoculi* on the basis of biochemical testing as well as by blast analysis against the GenBank database of an amplified and sequenced ribosomal RNA gene (partial 16S ribosomal RNA gene and 16 S-23S ribosomal RNA intergenic spacer; ISR) (details provided below). A subset of isolates collected by one of the authors (KAC) was also subject to analysis by MALDI-TOF testing (see below). Ocular swabs were streaked onto trypticase soy agar with 0.5 % sheep blood plates (SBA) and incubated at 35 °C. Colonies with morphology consistent with *Moraxella* spp. after 24–48 h of incubation were subcultured for further characterization. Isolates that were catalase-positive, oxidase-positive, Gram-negative coccobacilli, negative for carbohydrate fermentation, able to reduce nitrate, negative for casein hydrolysis, and able to deaminate phenylalanine were characterized as *M. bovoculi *. Isolates were stored frozen at −70 °C until use.

The isolates provided by KAC (*n*=59) were also subject to MALDI-TOF testing by incubating isolates overnight on SBA at 35 °C in a 5–10 % CO_2_ atmosphere. Isolates were tested according to the MALDI-TOF instrument manufacturer’s recommended procedure for the direct smear method using α-cyano-4-hydroxycinnamic acid (Bruker Daltronics, Billerica, MA, USA), and subjected to automatic detection in a positive linear mode between 2 kDa and 20 kDa m/hz, with a laser frequency of 60 Hz (Microflex LT MALDI-TOF MS, Bruker Daltronics). The system was calibrated for reference masses of 3637–16 952 Da using the manufacturer’s supplied bacterial test standard. Up to 240 spectrum profiles were obtained per colony, and all colonies were tested in duplicate. Bacterial identifications were determined using commercial software and the database provided by the manufacturer (Compass, 4.1, Bruker Daltronics). Identity scores >2.0 were considered very good to the genus and species level per the manufacturer’s guidelines.

### Genomic DNA and PCR

*M. bovoculi* isolates were thawed, streaked onto 5 % SBA, and incubated at 35 °C for 18–20 h. Genomic DNA was purified from whole bacteria using a commercial kit (DNEasy kit; Qiagen, Germantown, MD, USA). Further confirmation of all isolates as *M. bovoculi* was made by blast analysis of an amplified and sequenced ribosomal RNA gene (partial 16S ribosomal RNA gene and 16 S-23S ribosomal RNA intergenic spacer; ISR) against the GenBank database. This region was amplified from genomic DNA with primers ISRdown (5′-GTG AAG TCG TAA CAA GGT AGC CGT-3′) and ISRup (5′-ACC GAC GCT TAT CGC AGG CTA TCA-3′) using previously described PCR conditions [20]; all isolates had high % identify (99.6–100 %) to ISR sequences of *M. bovoculi* that were previously submitted to the GenBank database.

The *pilA* gene was amplified from genomic DNA using primers Mbovoc_Pilin_Dn (5′-GTG GGG TTA CAT AAA TAT AAA GA-3′) and Mbovoc_PilinUp3 (5′-GAT TAA TCA AAC CTT CAA ACA C-3′). These primers were designed to amplify a 685 bp fragment that spanned a type IV pilin (PilA) that was identified in the draft genome sequence of *M. bovoculi* 237 (GenBank accession no. AOMT01000037.1; locus_tag: MBO_08958; COG4969 Tfp pilus assembly protein, major pilin PilA; GenBank accession no. KDN24455 and [21]). These primers were located from 88 base pairs upstream of an ATG start codon to 138 base pairs downstream of a TAG stop codon. The PCR conditions were an initial denaturation at 95 °C for 1 min followed by 35 cycles of 95 °C for 30 s, 44 °C for 30 s, and 72 °C for 2 min, and a final incubation at 72 °C for 5 min. The PCR products were purified (QIAquick PCR Purification Kit, Qiagen) and sequenced at the UC Davis DNA Sequencing Laboratory (Davis, CA, USA). Final *pilA* and ISR gene sequences were determined from overlapping sequences assembled with DNA sequence analysis software (Sequencher 5.4.6, Gene Codes Corporation, Ann Arbour, MI, USA).

### Nucleotide sequence accession numbers

The ISR and *pilA* nucleotide sequence accession numbers for the 94 *M *. *bovoculi* evaluated for this study are MT353781-MT353874 (ISR sequences) and MT333648-MT333741 (*pilA* sequences). For comparing PilA sequences from this set of *M. bovoculi* isolates with previously reported pilin-related sequences in *M. bovis *, *M. bovoculi* and *M. ovis *, the deduced amino acid sequence of pilin- or PilA-related sequences for *M. bovis *, *M. bovoculi* and *M. ovis* were downloaded from the GenBank database. Accession numbers of sequences used for these comparisons are provided in Table 2.

**Table 2.** Source information and GenBank accession nos. for *M. bovis, M. bovoculi* and *M. ovis* pilin-related proteins

Description	Accession no.	Species	Strain	Length (aa)	Notes
prepilin	L32969	*bovis*	3W07	158	source: bovine pinkeye; Serogroup B
prepilin	L32965	*bovis*	218R	158	source: bovine pinkeye; Serogroup F
pilin; Tfp pilus assembly protein PilE	AAA53087	*bovis*	Dalton 2d	156	source: bovine pinkeye; Serogroup C
prepilin; Tfp pilus assembly protein, major pilin PilA	AAA53559	*bovis*	FL462	157	source: bovine pinkeye; Serogroup G
prepilin; Tfp pilus assembly protein, major pilin PilA	AAA53562	*bovis*	H358CS	159	source: bovine pinkeye; Serogroup D
prepilin	L32968	*bovis*	S276R	160	source: bovine pinkeye; Serogroup A
prepilin; Tfp pilus assembly protein, major pilin PilA	AAA53561	*bovis*	TAT849	159	source: bovine pinkeye; Serogroup E
type IV pilin PilA	KDN24455	*bovoculi*	237	152	source: bovine pinkeye; USA: California; culture collection: ATCC: BAA-1259
hypothetical protein AAX06_02925; pilin	AKG07295	*bovoculi*	22 581	156	source: bovine deep nasopharyngeal swab (asymptomatic animal); USA:Missouri
hypothetical protein AAX05_08035; pilin	AKG10099	*bovoculi*	23 343	156	source: bovine deep nasopharyngeal swab (asymptomatic animal); USA: Tennessee
hypothetical protein AAX07_08580; pilin	AKG12021	*bovoculi*	28 389	156	source: bovine deep nasopharyngeal swab (asymptomatic animal); USA: Kentucky
hypothetical protein AAX11_08125; pilin	AKG13989	*bovoculi*	33 362	156	source: bovine deep nasopharyngeal swab (asymptomatic animal); USA: Kansas
hypothetical protein AAX08_01970; Tfp pilus assembly protein, major pilin PilA	AKG14945	*bovoculi*	57 922	152	source: bovine pinkeye; USA: Kansas
hypothetical protein AAX09_01900	AKG18359	*bovoculi*	58 069	152	source: bovine pinkeye; USA: Nebraska
prepilin-type N-terminal cleavage/methylation domain-containing protein; Tfp pilus assembly protein, major pilin PilA	AKG16627	*bovoculi*	58 086	152	source: bovine pinkeye; USA: Virginia
prepilin-type N-terminal cleavage/methylation domain-containing protein	NSM11559	*bovoculi*	KZ-1	152	source: bovine eye; country of origin: Kazakhstan: Akmola region
pilin	WP_063514484	*ovis*		156	
pilin	WP_112744298	*ovis*		156	
hypothetical protein MOVS_07875; pilin	ANB91903	*ovis*	199/55	156	source: bovine pinkeye; country of origin: Norway; culture collection: ATCC: 33 078
Two subunits pilin	SPX85670	*ovis*	NCTC11019	156	contig: ERS1826247SCcontig000017
Two subunits pilin	STY87629	*ovis*	NCTC11227	156	contig: ERS1247844SCcontig000001
Two subunits pilin	STZ05528	*ovis*	NCTC11969	156	contig: 58901_D01158901_D01558901_D012

### Pilin sequence comparisons

The 94 *pilA* gene sequences were compared using a Muscle alignment (version 3.8.425 by Robert C. Edgar; Geneious Prime 2020.1.2). The deduced amino acid sequences of the 94 pilin sequences were aligned and compared with one another and to previously reported *M. bovis *, *M. ovis* and *M. bovoculi* pilin sequences (Table 2). Alignments were performed using Clustal Omega fast clustering (mBed algorithm in Geneious Prime 2020.1.2). Creation of a neighbour-joining consensus phylogenetic tree was performed using the Geneious Tree Builder (Jukes-Cantor genetic distance model; resampling: bootstrapping with 1000 replicates).

## Results

### DNA and deduced amino acid sequences of *M. bovoculi* pilin

**Fig. 1.**
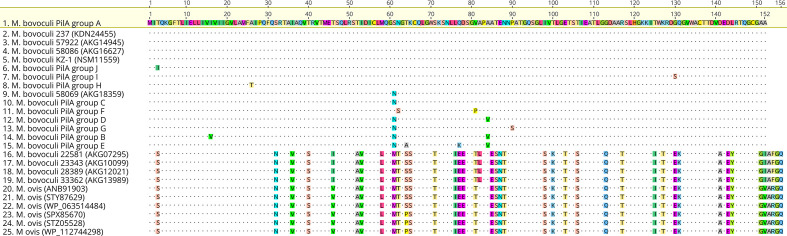
Alignment of the ten *M *. *bovoculi* PilA groups A–J deduced amino acid sequences (sequences 1, 6–8 and 10–15) identified in 94 *M. bovoculi* isolates derived from cattle with IBK that were evaluated for this study and previously reported *M. bovoculi* and *M. ovis* pilin-related sequences. Sequences 16–19 were from deep nasopharyngeal swabs of cattle without IBK that were first reported in [7]. Sequence 2 is derived from the whole-genome sequence of the type strain of *M. bovoculi *. Previously reported *M. ovis* pilin-related sequences 20–25 showed the most similarity to pilin-related sequences of *M. bovoculi* that were reported from deep nasopharyngeal swabs of IBK-asymptomatic cattle. Alignment created using Geneious version 2020.1 created by Biomatters; available from https://www.geneious.com.

A 459 bp ORF was identified in the sequenced amplicons of all 94 *M *. *bovoculi* isolates; 20 of these ORFs were unique. The deduced amino acid sequences of these 20 ORFs encoded ten unique PilA sequences that were designated *M. bovoculi* PilA groups A through J (Fig. 1). The number of isolates in these groups were 49, 19, 14, 5 and 2 for PilA groups A, B, C, D and E, respectively; one isolate each represented PilA groups F through J. The most frequently identified PilA sequence in this collection of samples was group A, which was identified in 22 of the 28 counties from which the 94 isolates originated (Table 3).

**Table 3.** Summary of year and county distribution of ten PilA groups identified in 94 isolates of *M. bovoculi* isolated from the eyes of cattle with IBK. Deduced amino acid sequences for each PilA group and associated GenBank accession numbers are shown below

	PilA group∗									
	A	B	C	D	E	F	G	H	I	J
**No. of isolates (no. of unique *pilA* DNA sequences)†**	49 (6)	19 (1)	14 (5)	5 (1)	2 (2)	1	1	1	1	1
**Year(s) isolated**	2002; 2006; 2007; 2008; 2009; 2010; 2011; 2012; 2013; 2014; 2015; 2016; 2017	2002; 2006; 2007; 2008; 2009; 2011; 2012; 2013; 2017	2006; 2007; 2008; 2009; 2010; 2012; 2013; 2015; 2017	2014; 2016; 2017	2011; 2017	2002	2015	2016	2002	2012
**Source county‡**	Chaves (NM); El Dorado; Franklin (ID); Fresno; Humboldt; Kern; Kings; Marin; Mendocino; Merced; Modoc; Placer; Plumas; San Bernardino; Siskiyou; Sonoma; Stanislaus; Tulare; Washoe (NV); Yavapai (AZ); Yolo; Yuba	Jerome (ID); Marin; Sacramento; San Joaquin; Stanislaus; Trinity; Yuba	Calaveras; Kern; Marin; Merced; Modoc; Sonoma; Stanislaus; Yolo; Yuba	Lassen; Kern; Merced	Sonoma; Kings	Yuba	Yolo	Tulare	Yuba	Merced

­
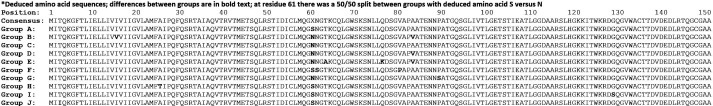

†**Genbank accession numbers:** Group A (MT333648; MT333649; MT333650; MT333651; MT333652; MT333654; MT333655; MT333657; MT333659; MT333660; MT333661; MT333662; MT333667; MT333669; MT333674; MT333694; MT333706; MT333733; MT333734; MT333737; MT333738; MT333740; MT333741; MT333689; MT333690; MT333691; MT333692; MT333697; MT333696; MT333712; MT333707; MT333709; MT333711; MT333715; MT333717; MT333700; MT333719; MT333721; MT333724; MT333730; MT333679; MT333676; MT333680; MT333682; MT333686; MT333687; MT333718; MT333720; MT333722); Group B (MT333656; MT333663; MT333664; MT333703; MT333708; MT333723; MT333668; MT333672; MT333675; MT333710; MT333714; MT333665; MT333666; MT333735; MT333695; MT333702; MT333698; MT333684; MT333685); Group C (MT333671; MT333688; MT333670; MT333732; MT333736; MT333739; MT333701; MT333705; MT333716; MT333683; MT333704; MT333725; MT333727; MT333681); Group D (MT333699; MT333678; MT333728; MT333729; MT333731); Group E (MT333693; MT333673); Group F (MT333653); Group G (MT333726); Group H (MT333677); Group I (MT333658); Group J (MT333713). Isolates corresponding to accession numbers MT333669 (PilA group A) and MT333668 (PilA group B) were isolated from the same calf (but different IBK-affected eyes) on 5-26-2007 and 6-25-2007, respectively. Isolates corresponding to accession numbers MT333675 (PilA group B) and MT333674 (PilA group A) were isolated from the same calf (but different IBK-affected eyes) on 6-5-2007 and 8-17-2007, respectively.

‡Counties located in California except where indicated.

The deduced amino acid sequences of these ten unique PilA sequences shared a high degree of sequence similarity with overall identical sites and pairwise identity of 92.8 and 98.1 %, respectively. Differences in deduced amino acid sequences between the ten groups included four conservative and eight radical amino acid replacements. At residue 61, the sequences were equally divided between those with serine versus asparagine residues. In one of the pairs of SFREC isolates from 2008 that originated from a single animal, one exhibited a PilA group A sequence (accession MT333669) while the second obtained from the opposite eye approximately 4 weeks later exhibited a group B sequence (MT333668). In the second pair of two isolates that originated from a single animal, the initial isolate exhibited a PilA group B sequence (accession MT333675), while the second isolate obtained from the opposite eye approximately 10 weeks later exhibited a PilA group A sequence (accession MT333674).

### Comparisons with previously reported *M. bovoculi, M. ovis* and *M. bovis* pilin-related sequences

**Fig. 2.**
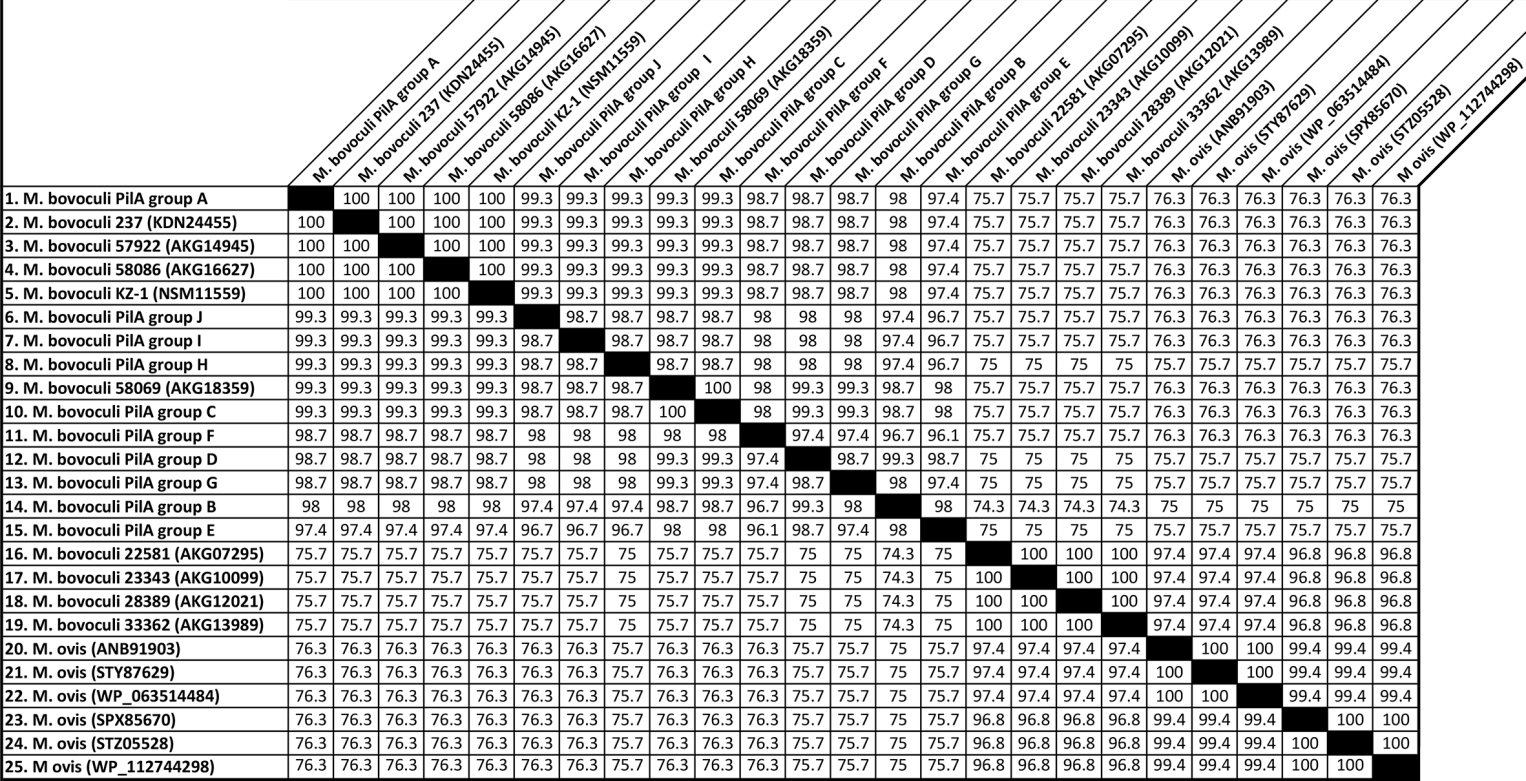
Similarity matrix showing percent identities between sequences depicted in Fig. 1 (created using Geneious version 2020.1 created by Biomatters; available from https://www.geneious.com).

Previously reported pilin-related sequences in *M. bovoculi* from IBK-affected cattle in Kansas (strain 57922), Virginia (strain 58086) and Kazakhstan (strain KZ-1) (respective GenBank accession nos.: AKG14945, AKG16627 and NSM11559) and the PilA protein from the type strain of *M. bovoculi* (237; KDN24455) were identical to the PilA group A deduced amino acid sequence. A previously reported hypothetical protein from a Nebraska isolate of *M. bovoculi* (strain 58069) from an IBK-affected cow (GenBank accession no.: AKG18359) was identical to the PilA group C deduced amino acid sequence. See Fig. 2.

Comparisons of the PilA groups A–J deduced amino acid sequences with putative pilin deduced amino acid sequences that were identified in *M. bovoculi* from deep nasopharyngeal swabs of cattle that did not have IBK (* M. bovoculi* strains 22 581, 23 343, 28 389 and 33 362 [7] with respective GenBank accession nos.: AKG07295, AKG10099, AKG12021 and AKG13989) revealed 74.3–75.7 % identity between sequence pairs (Fig. 2). Comparisons between previously reported *M. ovis* pilin sequences (GenBank accession nos.: WP_063514484, WP_112744298, ANB91903, SPX85670, STY87629 and STZ05528) and *M. bovoculi* PilA groups A–J showed approximately 75 % identity. These six *M *. *ovis* sequences exhibited 96.8–97.4 % identity to pilin-related sequences from *M. bovoculi* from deep nasopharyngeal swabs of IBK-asymptomatic cattle (strains 22 581, 23 343, 28 389 and 33 362 with respective GenBank accession nos.: AKG07295, AKG10099, AKG12021 and AKG13989) (Fig. 2).

**Fig. 3.**
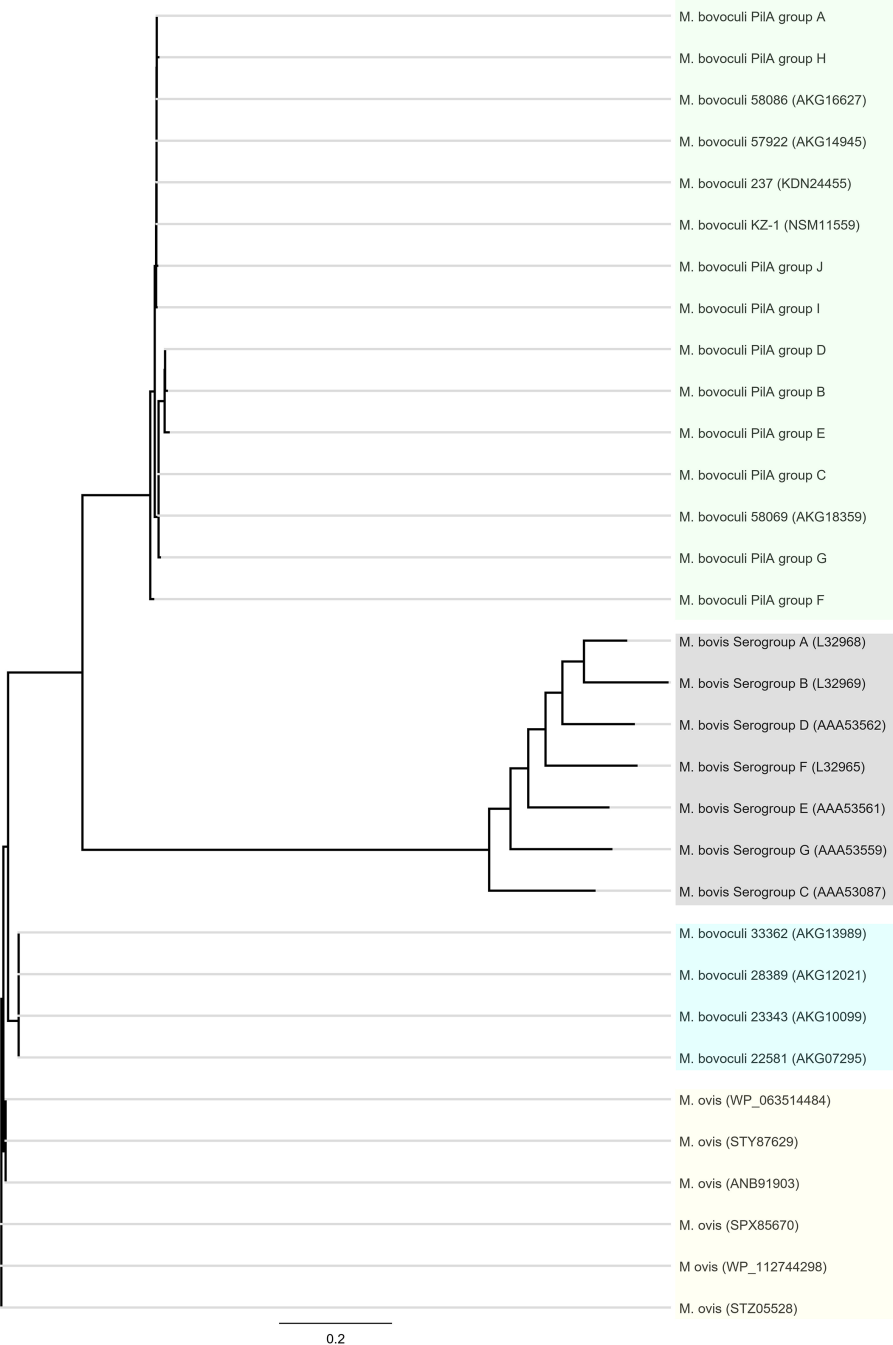
Unrooted neighbour-joining consensus tree depicting relationships between *M. bovoculi* PilA groups A–J characterized from 94 *M *. *bovoculi* isolates from cattle with IBK in the Western USA and pilin-related deduced amino acid sequences previously reported in *M. bovoculi* from cattle with IBK (green box), *M. ovis* (yellow box) and *M. bovis* (grey box). Blue box indicates previously characterized *M. bovoculi* that were isolated from the nasopharynx of IBK-asymptomatic cattle [7]. GenBank accession numbers are shown in parentheses. Bar, 0.2 substitutions per site (Geneious version 2020.1 created by Biomatters; https://www.geneious.com).

Amongst the deduced pilin amino acid sequences for the previously reported eight *M *. *bovis* pilin serogroups A, B, C, D, E, F and G (GenBank accession nos.: L32968 (serogroup A); L32969 (serogroup B); AAA53087 (serogroup C); AAA53562 (serogroup D); AAA53561 (serogroup E); L32965 (serogroup F); and AAA53559 (serogroup G) [22]) there was 61.3–78.1 % identity (Fig. 2). The percent identity between the deduced amino acid sequence of these *M. bovis* pilin serogroup sequences and *M. bovoculi* PilA group A–J sequences ranged from 29.5–32.3 %. A phylogenetic tree depicting relationships between the pilin sequences listed in Table 1 and the ten *M *. *bovoculi* PilA groups identified in this study showed distinct clustering of pilin-deduced amino acid sequences from seven defined *M. bovis* serogroups, *M. bovoculi* isolated from cattle with IBK, *M. ovis* and *M. bovoculi* isolated from the nasopharynx of cattle without IBK (Fig. 3).

## Discussion

In this study we found ten unique structural pilin (PilA)-deduced amino acid sequences amongst a collection of *M. bovoculi* that had been isolated from eyes of IBK-affected cattle throughout California and four other Western states (Idaho, Nevada, New Mexico and Arizona). Given the limited sample size and geographic distribution of isolates that we examined, however, it is impossible to say how likely or not it is that more PilA groups might exist amongst *M. bovoculi *. Since two of these PilA group sequences (A and C) matched PilA sequences in *M. bovoculi* from IBK-affected cattle from other locations in the USA (Kansas, Nebraska, Virginia) and Asia (Akmola region of Kazakhstan), it is possible that the PilA groups identified in this study are representative of *M. bovoculi* PilA sequences in general. Of the ten PilA groups that we identified, three (A, B and C) were the most widely distributed over geography and time, however, it is likely that a larger sample size would have revealed additional locations of these less well-represented PilA groups.

Among the ten PilA groups that were characterized in this study population, the overall degree of difference was very small compared to the seven *M *. *bovis* serogroups that have been characterized [17, 22]. Because *M. bovoculi* isolates that have been examined thus far via whole-genome sequencing have not displayed evidence for an I/Q pilin type phase shift [6] that was described in *M. bovis* [18, 23], it seems likely that any additional PilA group types that may be identified in the future will probably exhibit a relatively low degree of variability from one another as compared to the variability that is observed in *M. bovis* exhibiting different pilin serogroups.

It is currently not known whether or not PilA expression in *M. bovoculi* is a prerequisite for establishing colonization of the ocular surface. While published studies thus far have not supported a causal role for at least one strain of *M. bovoculi* in IBK [5], positive correlations have been reported between clinical signs of IBK and the presence of *M. bovoculi* [2, 3]. This suggests that *M. bovoculi* attachment to bovine ocular surfaces may be necessary for it to survive in/around ocular mucosal surfaces. If true, given what is known about the general role for pilins in host attachment and survival, it is logical to conclude that pilin probably does play a role in the ability of *M. bovoculi* to exist on ocular surfaces. Previous studies in other Moraxella species demonstrated that pilin is important for colonization and biofilm formation [24, 25] and a recent study demonstrated that *M. bovoculi* forms biofilms [26]. This suggests that pilin expression in *M. bovoculi* is likely involved in its ability to colonize the bovine eye. The fact that a putative PilA protein from *M. bovoculi* associated with the nasopharynx of IBK-asymptomatic cattle in Missouri, Tennessee, Kentucky and Kansas [7] and PilA from *M. bovoculi* from IBK-affected cattle exhibit only ~75 % identity between deduced amino acid sequences raises the possibility that differences in pilin sequence allow survival on different mucosal sites (for example, ocular surface versus nasopharynx).

Whether or not differences between the 10 PilA groups that we identified have any bearing on evasion of a host immune response during clinical IBK associated with the presence of *M. bovoculi* remains to be determined. For *M. bovis* strain Epp63 it is known that recombination events involving pilin-expressing genes allow different forms of pilin to be expressed [18], and that these different forms of pilin are associated with colonization versus maintenance of infection [27]. In the population of *M. bovoculi* isolates that we examined for this study we identified two PilA groups in each of the two animals (PilA groups A and B) where IBK developed in different eyes over a period of weeks. In one animal a PilA group A isolate was followed by identification of a PilA group B isolate, while in the other animal in the same herd the initial isolate was a PilA group B isolate followed by a group A isolate. Additional studies are needed to determine whether cattle develop immune responses to *M. bovoculi* pilin during ocular infections and whether the sorts of PilA group changes that we observed were reflective of host immune selection or just pure coincidence.

## Conclusion

*M. bovoculi* PilA deduced amino acid sequences exhibit some diversity, however, overall, PilA sequences are relatively conserved across geographically diverse isolates from cattle with IBK and much more conserved relative to *M. bovis* pilin serogroups. The exact role that *M. bovoculi* PilA might play in the ability of *M. bovoculi* to exist in/around bovine ocular tissues remains to be determined.
